# Ameloblastic fibrosarcoma of the maxilla arising in an old woman, a rare case report and literature review

**DOI:** 10.1186/s12903-024-04509-x

**Published:** 2024-06-27

**Authors:** Shiyue Liu, Hong Li, Youhong Dong, Dongdong Zhang

**Affiliations:** 1 Department of Oncology, Xiangyang No.1 People’s Hospital, Hubei Univeristy of Medicine, Jiefang Road No. 15, XiangYang, Hubei 441000 China; 2 Department of Rehabilitation Medicine, Xiangyang No.1 People’s Hospital, Hubei Univeristy of Medicine, XiangYang, Hubei China

**Keywords:** Ameloblastic fibrosarcoma, Odontogenic tumor, Maxillary bone, Surgical, Adjuvant radiotherapy

## Abstract

**Background:**

Ameloblastic fibrosarcoma (AFS) is a rare malignant odontogenic tumor, commonly occurring in young adults and typically affecting the mandibular region. We report an exceptionally rare and highly atypical case of AFS in an elderly female patient originating from the maxillary bone.

**Case presentation:**

A 66-year-old woman was admitted with a two-week history of a lump in her left upper molar. CT scans suggested a cyst in the maxillary bone. An incisional biopsy revealed a spindle cell neoplasm. MRI showed abnormalities in the left maxilla, indicating a possible tumorous lesion. The patient underwent a subtotal maxillectomy, wide tumor excision, intraoral epithelial flap transplantation, and dental extraction. Histology identified atypical tumor cells with visible mitotic figures. Immunohistochemistry showed negative for PCK and CD34 expression, but positive for Vimentin and SMA expression. The Ki-67 proliferation index ranged from 30 to 50%. These findings suggested a potentially malignant soft tissue tumor in the left maxilla, leaning towards a diagnosis of AFS. The patient received postoperative radiotherapy. There was no recurrence during the six-month follow-up.

**Conclusion:**

Based on repeated pathological evidence, we report a rare case of an elderly female with AFS originating from the maxillary bone. Surgery and postoperative radiotherapy resulted in a favorable outcome.

**Supplementary Information:**

The online version contains supplementary material available at 10.1186/s12903-024-04509-x.

## Introduction

Ameloblastic fibrosarcoma (AFS) is a rare odontogenic neoplasm, accounting for less than 5% of all odontogenic tumors, characterized by distinct cytologic atypia, increased cellularity with a diminished epithelial component, and invasive behavior [1]. The first case of AFS was reported in 1887 [2]. In 2005, the World Health Organization (WHO) classified odontogenic sarcomas into two entities: AFS and ameloblastic fibro-odontosarcoma/fibrodentino-sarcoma (AFOS/AFDS) [3]. However, according to the 2017 and 2022 WHO Classification of Head and Neck Tumors, the odontogenic sarcoma was listed as a subtype of malignant odontogenic tumors, without further subdivision [4, 5].

AFS typically manifests most frequently during the second and third decades of life, exhibiting a broad age spectrum. The mandible stands out as the predominant site of occurrence in a majority of cases [6]. It was postulated that AFS represents the malignant counterpart of ameloblastic fibroma (AF) [7]. The tumor can either arise from a pre-existing AF or present de novo [8]. Due to the rarity of case reports, the specific pathogenesis remains elusive, and no uniform diagnostic and treatment standards have yet been established. In this study, an exceedingly rare case of AFS was reported, featuring involvement in the maxilla of an elderly woman.

## Case presentation

**Fig. 1:**
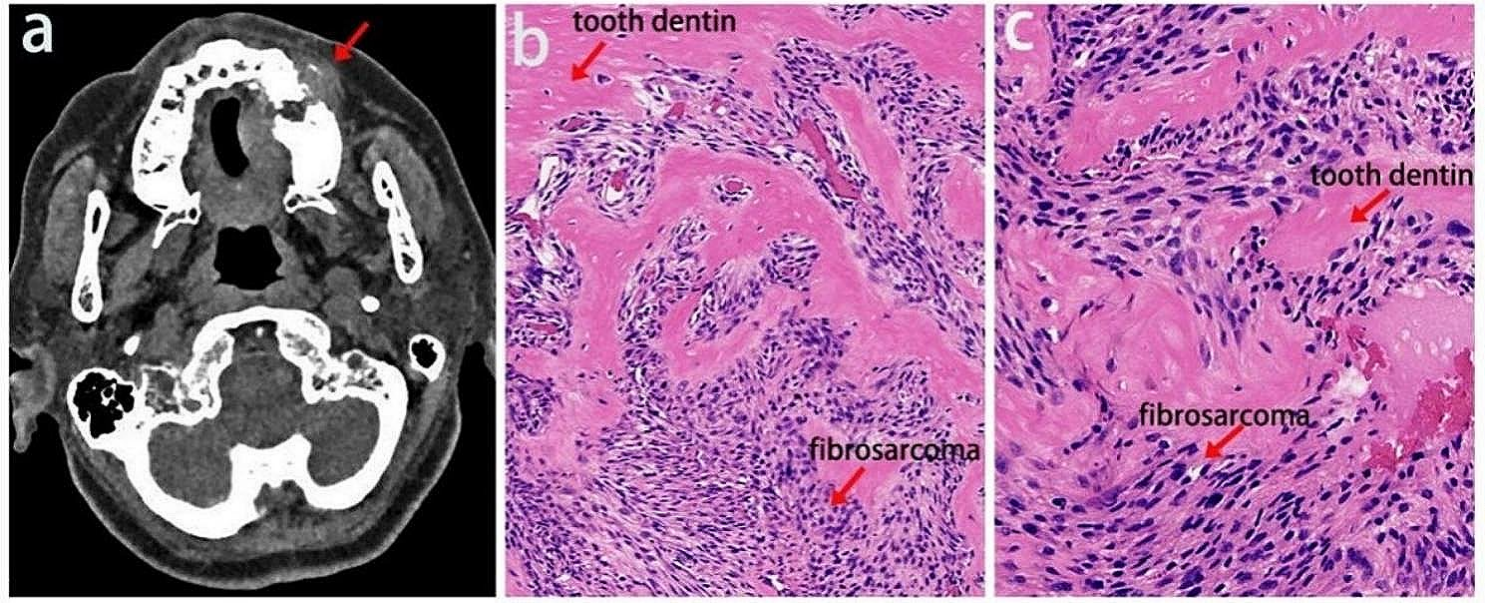
Maxillary CT and representative histopathologic features of malignant odontogenic tumors. (**a**) CT scan showed a mass in the left maxilla. (**b**) Fibrosarcoma under HE staining (Scale bars, 400 μm for 40 × magnification). (**c**) The stromal components consist of oval and spindle-shaped cells as well as plump and spindle-shaped matrix cells, showing increased cell abundance, nuclear polymorphism, cytological atypia, mitotic figures, and malformation (Scale bars, 200 μm for 100×magnification)

A 66-year-old female patient was admitted to our hospital with a chief complaint of a lump in the left upper molar area since two weeks before her admission to the hospital. The patient noticed the lump incidentally and described numbness in the area. She experienced a sour and painful sensation while biting down on her teeth. Upon examination, a mass measuring approximately 4.0 cm × 3.5 cm was found in the left upper molar region. The mass was firm on palpation with indistinct borders. No enlarged lymph nodes were identified, and normal occlusion was noted. Other examinations of the head, neck, and general physical assessment showed no abnormality. The maxillary computed tomography (CT) scan indicated the possibility of a maxillary bone cyst, with differential diagnoses such as odontogenic ameloblastoma and fibrous dysplasia pending further investigation (Fig. 1a). Subsequently, incisional biopsy was performed on the maxillary lesion to make a definitive diagnosis. The procedure revealed profound bone destruction in the maxilla beneath the gum flaps, accompanied by a substantial amount of granulation-like tissue. The tissue presented as brittle with indistinct borders. The hematoxylin and eosin (HE) staining were indicative of a spindle cell neoplasm within the soft tissues (Fig. 1b, c).

**Fig. 2:**
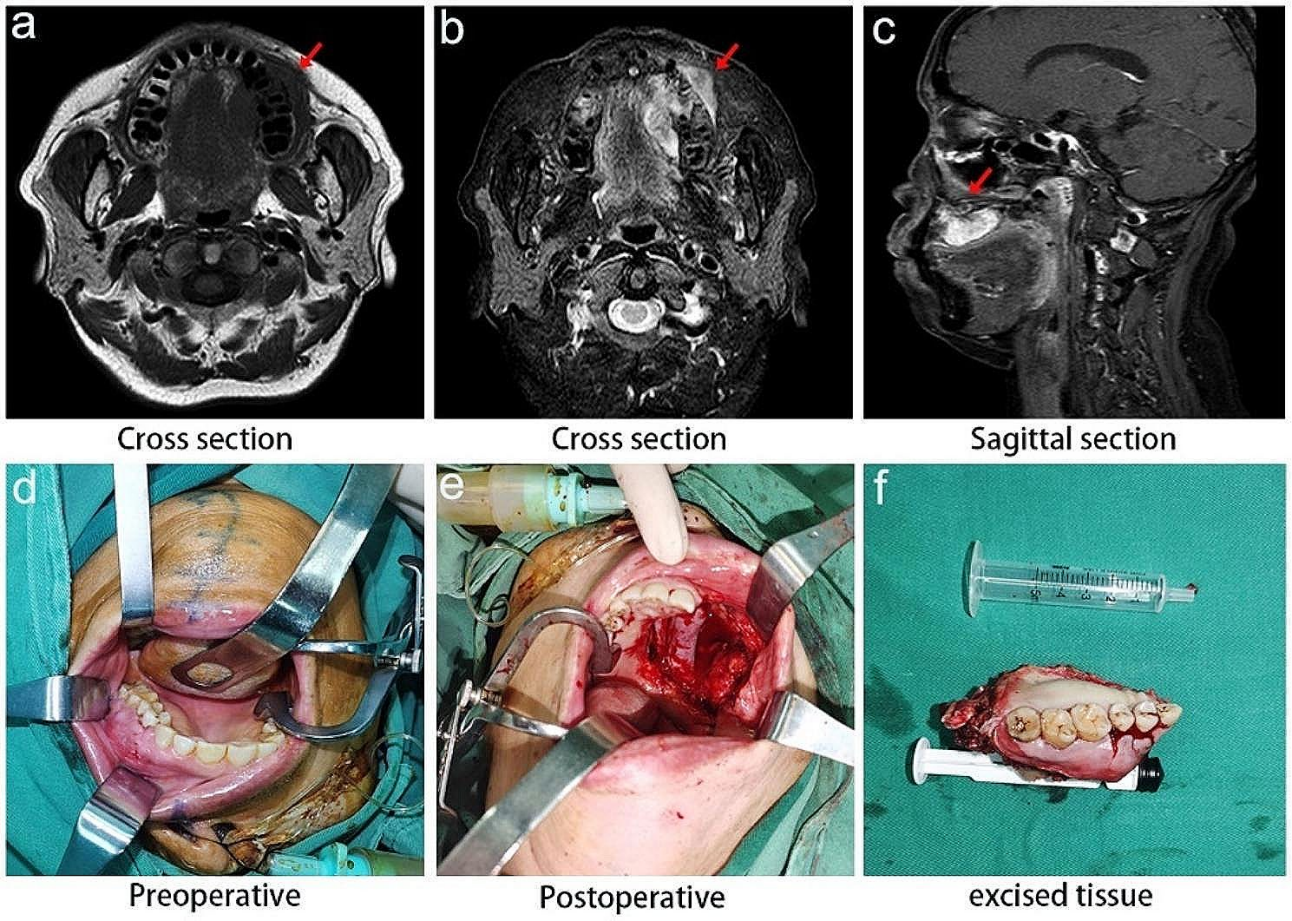
MRI imaging of the mass in the left maxilla and macroscopic findings with intraoperative observations of the tumor. (**a**) and **(b**) Cross-sectional MRI of the maxillary mass. (**c**) Sagittal section imaging of the maxillary mass under MRI. (**d**) Intraoral image of the tumor before surgery. (**e**) Intraoral image of the tumor after surgery. (**f**) The surgical maxillary fragment

Following this, magnetic resonance imaging (MRI) with maxillary enhancement was conducted, revealing anomalous alterations in the left maxilla and its adjacent soft tissues, measuring approximately 27 mm × 37 mm. No metastasis was observed in the head region. The findings led to the consideration of a tumorous lesion, as illustrated in Fig. 2a-c.

Further evaluations, including thoracic CT, electrocardiogram, echocardiography, maxillary CT scan, abdominal CT, and superficial lymph node ultrasound, were carried out. No surgical contraindications were identified. On June 15, 2023, the patient underwent subtotal maxillectomy, wide excision of the malignant tumor in the maxilla, intraoral epithelial flap transplantation, and dental extraction. The surgical procedure involved an extended incision around the left maxillary mass, exposing the bone surface after dissecting through the mucosa and submucosal tissues. Tooth #22 was subsequently extracted using forceps, confirming intact root apices. High-speed turbine and bone knives were utilized to access the maxilla, ensuring complete removal of the diseased tissue in the left maxilla. A portion of the surrounding tissue was subsequently sent for biopsy. No clear tumor involvement was observed within the inner, outer, anterior, posterior, or maxillary sinus floor margins. The preoperative and postoperative conditions are displayed in Fig. 2d-f.

**Fig. 3:**
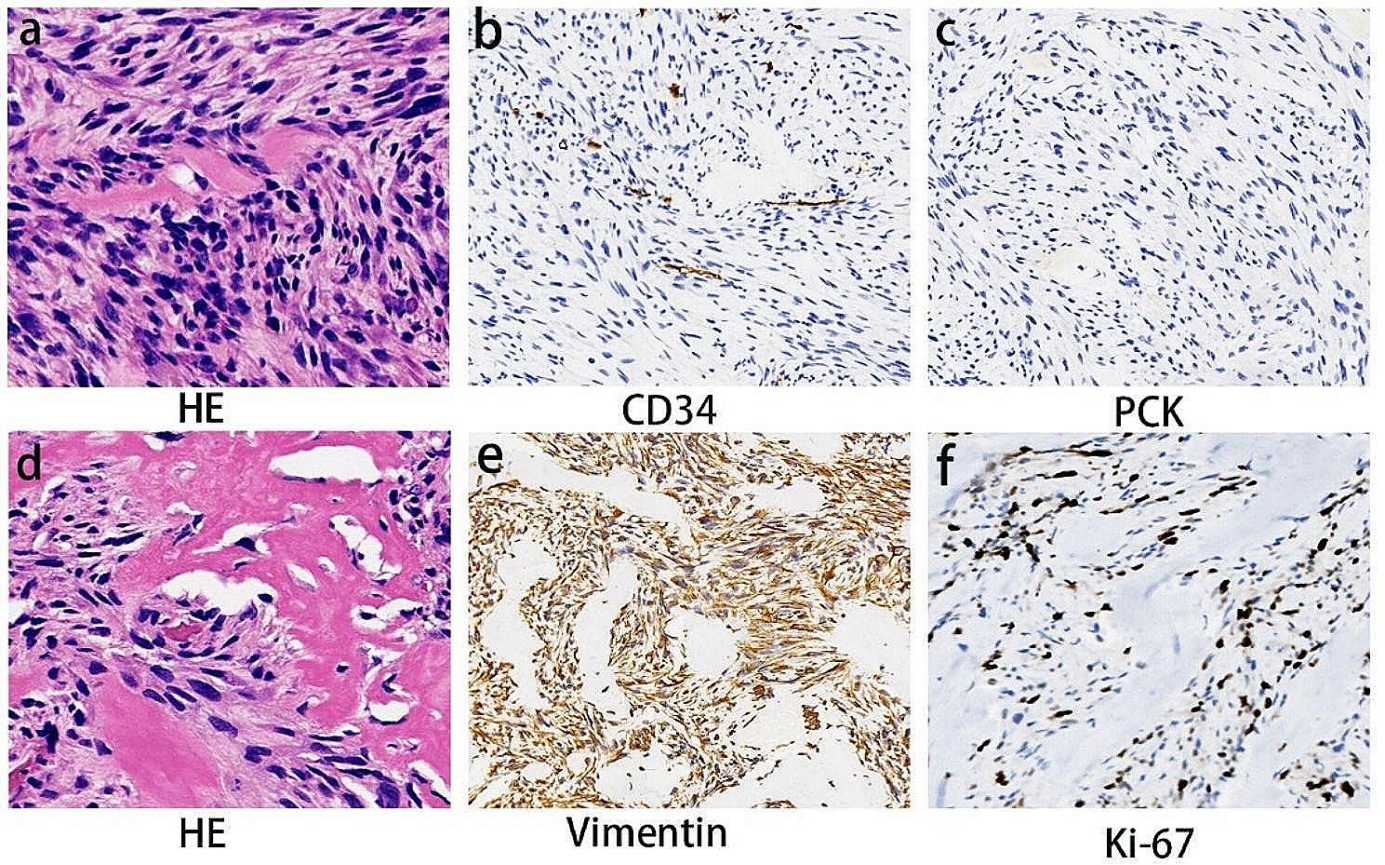
Histopathological findings following surgical resection of the maxillary lesion. (**a)** and (**d**) Histology of AFS revealed under HE staining (Scale bars, 100 μm for 200 × magnification). Negative reactivity to CD34 (**b**) and PCK (**c**). (**e**) Positive reactivity to Vimentin. (**f**) Reactivity to Ki-67 in the mesenchymal component with a labeling index of 30–50%. Scale bars, 200 μm for 100×magnification

The postoperative HE staining revealed that atypical tumor cells were arranged in bundles or woven patterns, with visible nuclear mitotic figures. The majority of the cells were fibrosarcoma cells interspersed with stromal components including enamel or dentin-like material, and within the fibrosarcoma background, there was an expression of P63-positive odontogenic epithelial component (Fig. 3a, d and S1). The epithelial tissue appeared benign, while the connective tissue component was malignant, indicating an increased cellularity, variation in nuclear size and shape, and evident mitotic activity. Dentin or enamel-like structures were possibly found within the stroma. Immunohistochemistry (IHC) results indicated that tumor cells exhibited negative expression levels for PCK and CD34, while being positive for Vimentin and SMA. Additionally, the Ki-67 proliferation index ranged from 30 to 50% (Fig. 3b, c, e, f). According to the integration of histological morphology and immunophenotyping, the diagnosis suggested a potential malignant soft tissue tumor in the left maxilla, with a greater proclivity towards AFS. The postoperative tumor staging for the patient is pT2N0M0, stage IIB. However, given the rarity of this case, it was recommended that the patient pursue further pathological consultation at a more advanced institution. Regrettably, due to financial constraints, the patient declined this recommendation.

**Fig. 4:**
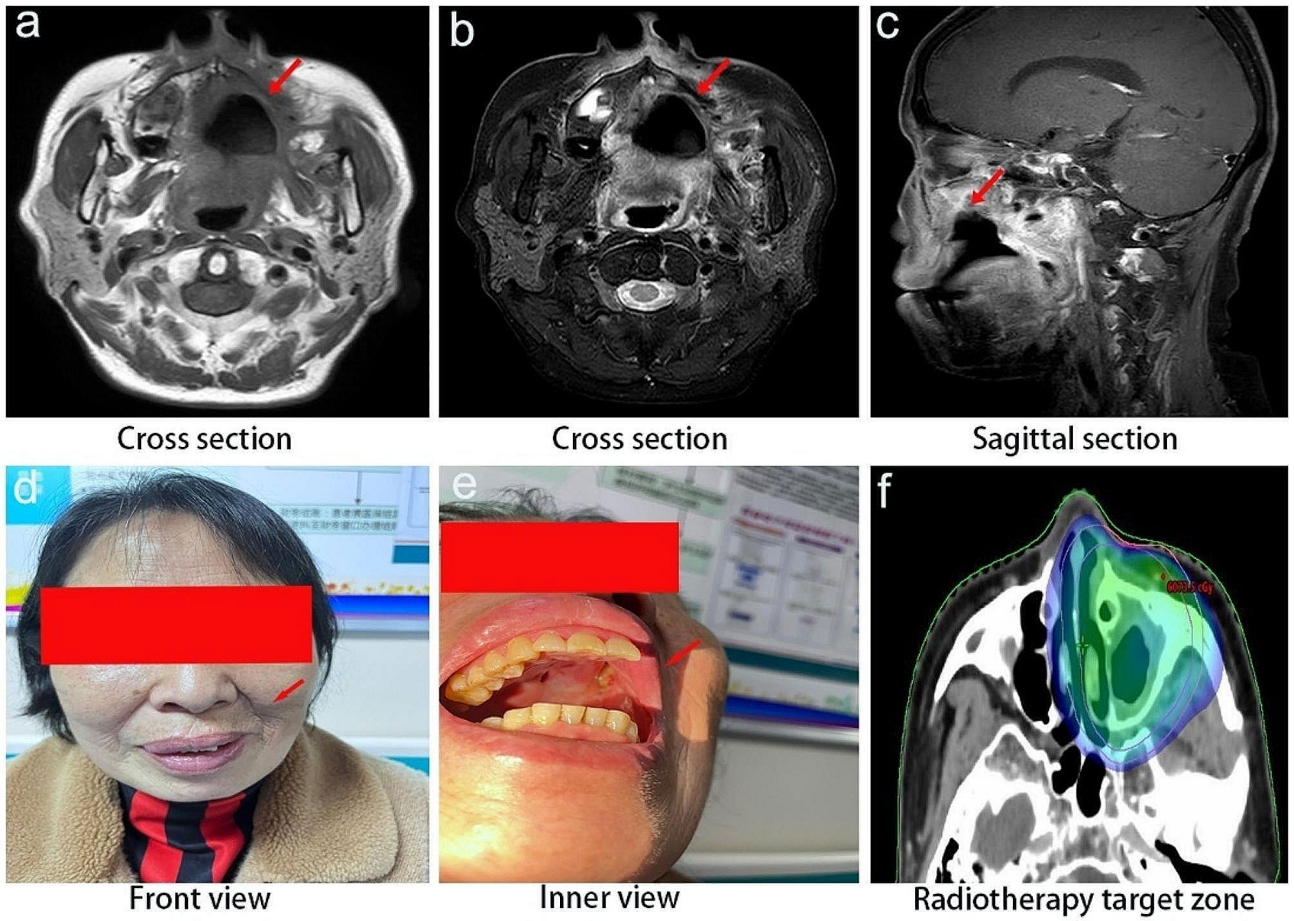
Postoperative patient follow-up data. (**a**) and (**b**) Cross-sectional MRI images after surgery. (**c**) Sagittal section MRI imaging after surgery. (**d**) and (**e**) Front view and intraoral view of the patient at 6-month follow-up after surgery. (**f**) Image illustrating the range of the radiation therapy target area post-surgery

One month after surgery, the patient received postoperative radiotherapy with a prescribed dose of 60 Gy/30f to the planning gross tumor volume (pGTV) and 54 Gy/30f to the planning target volume (PTV), as illustrated in Fig. 4f. The patient was recovered well after undergoing surgery and adjuvant radiotherapy, and no recurrence during 6-month follow-up was recorded. The current oral condition of the patient postoperatively is shown in Fig. 4a-e.

## Discussion

AFS represents a rare odontogenic tumor, with approximately 80% of cases originating in the mandible and a median onset age around 27 years [9–11]. In this study, an exceedingly infrequent case of AFS was reported, involving an elderly female patient, manifesting in the maxilla.

Similar to numerous instances of soft tissue sarcomas, the precise mechanisms underlying the development of AFS remain elusive. Emerging research indicated that AFS may be linked to genetic mutations, hereditary factors, inflammatory processes, traumatic events, and intricate epithelial-stromal interactions [12]. Prior research reported a correlation between AFS and loss of heterozygosity (LOH) in the short arms of chromosomes 3 and 9 [13]. Bcl-2 alteration may also participate in the pathogenesis of this neoplasm [14]. Comprehensive genomic testing in AFS patients revealed the presence of EGFR exon 20 insertions and MDM2 amplification, emerging as potential drivers of AFS development [9].

The diagnosis of AFS solely based on radiographic evidence is not highly reliable. Radiologically, AFS appears as a nebulous translucent mass, leading to occasional misdiagnoses as a cyst, as evidenced in the present case, wherein the initial CT scan led to an interpretation of a cyst in the patient [6]. However, further pathological confirmation could solidify the diagnosis of AFS.

Some studies have suggested that AFS can arise de novo or from preexisting benign lesions, such as AF, immature enamel cell fibroma, or odontoma [15]. While studies have mainly concentrated on differentiating AFS from AF [6], this case highlighted the importance of distinguishing AFS from AFOS. The nature and relationship between mixed odontogenic tumors and related lesions remain elusive. The main distinction between AFS and AFOS lies in the presence or absence of dental hard tissue components within the stroma [16]. In the current case, the primary consideration leaned towards AFS or AFOS, while subsequent pathological examination confirmed a higher likelihood of AFS due to the absence of dental hard tissue components within the stroma. Despite recommendations from experts that the presence or absence of dental hard tissue in the stroma does not impact treatment decisions, no strong inclination was found for external pathological consultation when the patient refused the recommendation [17, 18]. Furthermore, reflection on the diagnostic challenges encountered with AFS and AFOS, which could be attributed to the involvement of multiple stages in tooth development, including growth, calcification, and eruption [19]. Each stage encompasses different processes and introduces uncertainties under microscopic observations, posing challenges for accurate diagnosis. While some studies have reported that an abnormal CD34 expression level in the maxillary bone can assist in diagnosing AFS, in this particular case, CD34 expression level was negative, providing valuable information regarding the diagnosis and characteristics of this rare tumor [17]. AFS and AFOS typically exhibit positive immunostaining for Vimentin, indicating the presence of mesenchymal components.

Given the scarcity of data and the lack of comprehensive guidelines, there is currently no universally accepted treatment protocol for AFS. AFS is characterized by a low likelihood of distant metastasis, and it has exhibited a significant recurrence rate of up to 37% and a mortality rate of 19% [20]. For such cases, the preferred treatment strategy involves early and aggressive management utilizing surgical intervention accompanied by adjuvant radiotherapy. Administering high-dose radiation therapy directly to the tumor site has the potential to effectively lower the recurrence rate and inhibit tumor metastasis [21]. Nevertheless, when considering the use of radiation therapy in younger patients, it is crucial to strike a balance between the benefit of reducing local recurrence and the potential risk of long-term development of secondary malignancies [22]. A previous study reported that some pediatric patients with AFS have exhibited a favorable response to chemotherapy [23]. With the advancements in molecular targeted therapy, studies have also indicated that patients with BRAF or NTRK mutations may benefit from the use of targeted inhibitors, leading to the improved survival outcomes in some AFS patients [24, 25]. However, further research is essential to explore precision medicine approaches and enhance our understanding of the biological aspects of AFS.

In conclusion, a rare case of AFS in the maxilla of an elderly patient was reported. The diagnostic process and treatment experience were discussed. This case not only contributes to enriching the AFS database, but also may provide insights for the future research on AFS treatment.

### Electronic supplementary material

Below is the link to the electronic supplementary material.

Supplementary Material 1

## Data Availability

The clinical data supporting the conclusions of this manuscript will be made available by the authors.

## Abbreviations

CT: Computed tomography
MRI: Magnetic resonance imaging
AFS: Ameloblastic fibrosarcoma
AFOS: ameloblastic fibro-odontoma
pGTV: planning gross tumor volume
PTV: planning target volume
HE: Hematoxylin and eosin
IHC: Immunohistochemistry
AF: ameloblastic fibroma

## References

[1] Song Z, Yu C, Song X, Wei L, Liu A (2011). Primary solitary fibrous tumor of the thyroid - report of a case and review of the literature. J Cancer.

[2] Heath C. Lectures on certain diseases of the Jaws. Br Med J. 1887;1(1380):1257–61. 10.1136/bmj.1.1380.1257 PMC2534786 20751936

[3] Sciubba JJ, Eversole LR, Slootweg PJ. Odontogenic/ameloblastic carcinomas. *pathology & genetics of head & neck tumours* 2005.

[4] Vered M, Wright JM (2022). Update from the 5th Edition of the World Health Organization Classification of Head and Neck tumors: odontogenic and maxillofacial bone tumours. Head Neck Pathol.

[5] Sarradin V, Siegfried A, Uro-Coste E, Delord JP (2018). [WHO classification of head and neck tumours 2017: main novelties and update of diagnostic methods]. Bull Cancer.

[6] Loya-Solis A, Gonzalez-Colunga KJ, Perez-Rodriguez CM, Ramirez-Ochoa NS, Cecenas-Falcon L, Barboza-Quintana O. Ameloblastic fibrosarcoma of the mandible: a case report and brief review of the literature. *Case Rep Pathol* 2015, 2015:245026. 10.1155/2015/245026 PMC4377457 25861504

[7] Chrcanovic BR, Brennan PA, Rahimi S, Gomez RS (2018). Ameloblastic fibroma and ameloblastic fibrosarcoma: a systematic review. J Oral Pathol Med.

[8] Mohsenifar Z, Behrad S, Abbas FM (2015). Epithelial dysplasia in Ameloblastic Fibrosarcoma arising from recurrent Ameloblastic Fibroma in a 26-Year-old Iranian man. Am J Case Rep.

[9] Terada K, Yamada Y, Ishida Y, Yamamoto T, Kikuchi M, Nakashima Y, Haga H (2022). Ameloblastic fibrosarcoma of the maxilla with EGFR exon 20 insertions: relevance of whole-exome sequencing in molecular understanding and therapeutic proposals for rare cancers. Auris Nasus Larynx.

[10] Gilani SM, Raza A, Al-Khafaji BM (2014). Ameloblastic fibrosarcoma: a rare malignant odontogenic tumor. Eur Ann Otorhinolaryngol Head Neck Dis.

[11] Bregni RC, Taylor AM, Garcia AM (2001). Ameloblastic fibrosarcoma of the mandible: report of two cases and review of the literature. J Oral Pathol Med.

[12] Agaimy A, Skalova A, Franchi A, Alshagroud R, Gill AJ, Stoehr R, Baumhoer D, Bauer S (2020). Ameloblastic fibrosarcoma: clinicopathological and molecular analysis of seven cases highlighting frequent BRAF and occasional NRAS mutations. Histopathology.

[13] Galvao CF, Gomes CC, Diniz MG, Vargas PA, de Paula AM, Mosqueda-Taylor A, Loyola AM, Gomez RS (2012). Loss of heterozygosity (LOH) in tumour suppressor genes in benign and malignant mixed odontogenic tumours. J Oral Pathol Med.

[14] Pontes HA, Pontes FS, Silva BS, Cury SE, Fonseca FP, Salim RA, Pinto Junior Ddos S (2010). Immunoexpression of Ki67, proliferative cell nuclear antigen, and Bcl-2 proteins in a case of ameloblastic fibrosarcoma. Ann Diagn Pathol.

[15] Lai J, Blanas N, Higgins K, Klieb H (2012). Ameloblastic fibrosarcoma: report of a case, study of immunophenotype, and comprehensive review of the literature. J Oral Maxillofac Surg.

[16] Wang S, Shi H, Wang P, Yu Q (2011). Ameloblastic fibro-odontosarcoma of the mandible: imaging findings. Dentomaxillofac Radiol.

[17] Lee OJ, Kim HJ, Lee BK, Cho KJ (2005). CD34 expressing ameloblastic fibrosarcoma arising in the maxilla: a new finding. J Oral Pathol Med.

[18] Mainenti P, Oliveira GS, Valerio JB, Daroda LS, Daroda RF, Brandao G, Rosa LE (2009). Ameloblastic fibro-odontosarcoma: a case report. Int J Oral Maxillofac Surg.

[19] Reboucas PRM, Alencar CRB, Arruda M, Lacerda RHW, Melo DP, Bernardino IM, Bento PM (2021). Identification of dental calcification stages as a predictor of skeletal development phase. Dent Press J Orthod.

[20] Al Shetawi AH, Alpert EH, Buchbinder D, Urken ML (2015). Ameloblastic Fibrosarcoma of the Mandible: a Case Report and a review of the literature. J Oral Maxillofac Surg.

[21] Leider AS, Nelson JF, Trodahl JN (1972). Ameloblastic fibrosarcoma of the jaws. Oral Surg Oral Med Oral Pathol.

[22] Pillay RR, Bilski A, Batstone M (2016). Ameloblastic Fibrosarcoma arising in the Maxilla. Ochsner J.

[23] Gatz SA, Thway K, Mandeville H, Kerawala C, MacVicar D, Chisholm J (2015). Chemotherapy responsiveness in a patient with multiply relapsed ameloblastic fibro-odontosarcoma of the maxilla. Pediatr Blood Cancer.

[24] Kheder ES, Hong DS (2018). Emerging targeted therapy for tumors with NTRK Fusion Proteins. Clin Cancer Res.

[25] Zaman A, Wu W, Bivona TG. Targeting Oncogenic BRAF: past, Present, and Future. Cancers (Basel) 2019, 11(8). 10.3390/cancers11081197 PMC6721448 31426419

