# Supplementary figures and images for: Ameloblastic fibrosarcoma of the maxilla arising in an old woman, a rare case report and literature review
Source: BMC Oral Health. 2024 Jun 27;24:743. doi: 10.1186/s12903-024-04509-x (PMC11212157; doi:10.1186/s12903-024-04509-x)

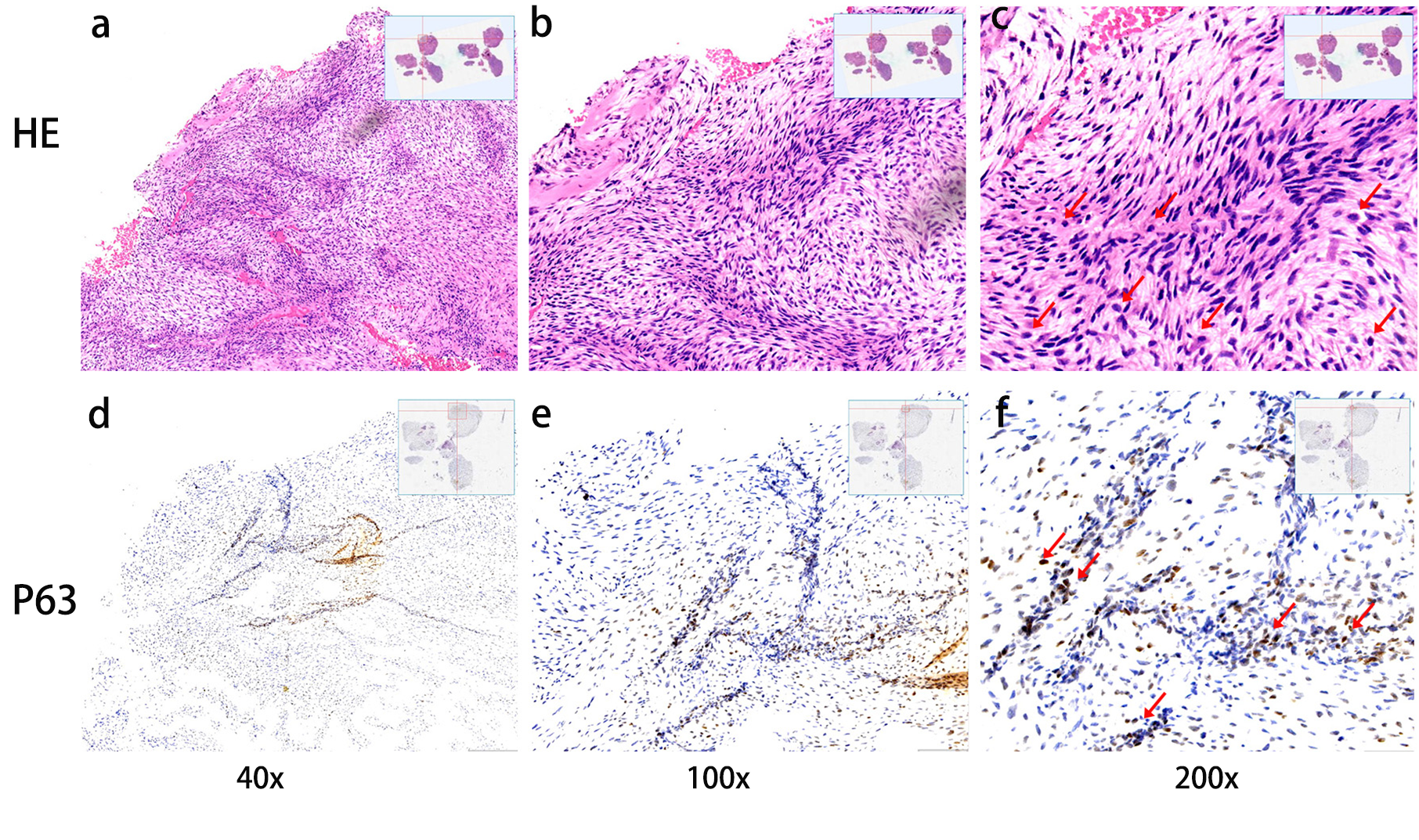

Supplement: Supplementary file 1 — Supplementary Material 1 [file 12903_2024_4509_MOESM1_ESM.tif]
